# In Vitro Pharmacological Modulation of PIEZO1 Channels in Frontal Cortex Neuronal Networks

**DOI:** 10.3390/brainsci14030223

**Published:** 2024-02-27

**Authors:** Pegah Haghighi, Mandee K. Schaub, Adam H. Shebindu, Gayathri Vijayakumar, Armaan Sood, Rafael Granja-Vazquez, Sourav S. Patnaik, Caroline N. Jones, Gregory O. Dussor, Joseph J. Pancrazio

**Affiliations:** 1 Department of Bioengineering, University of Texas at Dallas, Richardson, TX 75080, USA; heritier.adam@utdallas.edu (A.H.S.); sourav.patnaik@utsouthwestern.edu (S.S.P.); caroline.jones@utdallas.edu (C.N.J.); joseph.pancrazio@utdallas.edu (J.J.P.); 2 Department of Neuroscience, University of Texas at Dallas, Richardson, TX 75080, USA; mandee.schaub@utdallas.edu (M.K.S.); gayathri.vijayakumar@utdallas.edu (G.V.); armaan.sood@utdallas.edu (A.S.); gregory.dussor1@utdallas.edu (G.O.D.); 3 Center for Advanced Pain Studies, University of Texas at Dallas, Richardson, TX 75080, USA

**Keywords:** PIEZO1 channels, PIEZO1 antagonist GsMTx4, calcium imaging, cortical neurons, extracellular recording, mechanosensitive, multielectrode array, patch-clamp, spike

## Abstract

PIEZO1 is a mechanosensitive ion channel expressed in various organs, including but not limited to the brain, heart, lungs, kidneys, bone, and skin. PIEZO1 has been implicated in astrocyte, microglia, capillary, and oligodendrocyte signaling in the mammalian cortex. Using murine embryonic frontal cortex tissue, we examined the protein expression and functionality of PIEZO1 channels in cultured networks leveraging substrate-integrated microelectrode arrays (MEAs) with additional quantitative results from calcium imaging and whole-cell patch-clamp electrophysiology. MEA data show that the PIEZO1 agonist Yoda1 transiently enhances the mean firing rate (MFR) of single units, while the PIEZO1 antagonist GsMTx4 inhibits both spontaneous activity and Yoda1-induced increase in MFR in cortical networks. Furthermore, calcium imaging experiments revealed that Yoda1 significantly increased the frequency of calcium transients in cortical cells. Additionally, in voltage clamp experiments, Yoda1 exposure shifted the cellular reversal potential towards depolarized potentials consistent with the behavior of PIEZO1 as a non-specific cation-permeable channel. Our work demonstrates that murine frontal cortical neurons express functional PIEZO1 channels and quantifies the electrophysiological effects of channel activation in vitro. By quantifying the electrophysiological effects of PIEZO1 activation in vitro, our study establishes a foundation for future investigations into the role of PIEZO1 in neurological processes and potential therapeutic applications targeting mechanosensitive channels in various physiological contexts.

## 1. Introduction

PIEZO1 is a mechanosensitive cation channel expressed in various organs, including but not limited to the brain, heart, lungs, kidneys, bones, and skin [1]. Studies on PIEZO1 channels have revealed a trimeric structure resembling a propeller blade. This structure features an ion penetration hole at its center and is capped at the top by the C-terminal extracellular domain. High-speed atomic force microscopy has demonstrated that the application of external forces can flatten the naturally curved shape of PIEZO1 [2,3]. This flattening process is crucial, as it facilitates ion movement through the ion penetration site, contributing to the overall functionality of PIEZO1 as a mechanically activated ion channel. The roles of PIEZO1 include mechano-transduction-related processes such as embryonic vascular maturation, blood pressure regulation, and neural stem cell fate determination [4]. While it was initially reported that PIEZO1 was primarily expressed in non-excitable cells [1,5], PIEZO1 has been found to have roles in the nervous system, including axon regeneration [6], axonal guidance [7], axonal growth and pathfinding [8], and responsiveness of *Xenopus* retinal ganglion cell axons to their local mechanical environment [9]. In the mammalian cortex, PIEZO1 has been implicated in anterior cingulate cortical (ACC) neurons [10], cerebral cortex [11], frontal and motor cortex [12,13], astrocyte [11,14], microglia [15], capillary [16], and oligodendrocyte [17] signaling.

To date, limited work has been performed on the electrophysiological effects of PIEZO1 activation in cortical neurons. Three recent calcium imaging studies have reported a role for PIEZO1 in the excitatory effects of ultrasound in rodent cortical neurons [13,18,19]. Intracellular calcium dynamics include both calcium entry and calcium release from intracellular stores such as mitochondria and endoplasmic reticulum.

Prior work has shown that electrically active cells, including cortical neurons, can be readily cultured on MEAs for a wide range of applications [20,21,22,23,24,25]. MEAs resolve and record individual extracellular action potentials or spikes [26]. Complementary to single cell patch-clamp electrophysiology, extracellular action potentials are recorded noninvasively, enabling long-term and/or repeated monitoring of large cell populations. MEAs enable high-content quantitative measures of cellular excitability, such as spike rate, amplitude, bursting rates, and neuron firing synchronization [27]. 

In this study, we investigated the expression of PIEZO1 channels using immunocytochemistry in cultured neuronal networks derived from embryonic murine cortical tissue. Subsequently, the pharmacology of PIEZO1 was examined to understand how the inhibition and activation of this ion channel class modulates in vitro network activity. PIEZO1 can be modulated pharmacologically by GsMTx4, a spider venom peptide inhibitor [28], and Yoda1, a selective channel agonist [29], which effectively reduces the mechanical threshold for channel activation [30]. MEA recordings showed that Yoda1 transiently enhances the MFR of single units, while GsMTx4 inhibits both spontaneous activity and Yoda1-induced activity increase in neuronal networks. Interestingly, in a fraction of the recordings, there is a transient shift in the peak-to-peak amplitude in the recorded spikes with exposure to Yoda1, which we interpret in the context of known effects of mechanical stimulation on neuronal action potentials. To complement the MEA data, we conducted calcium imaging and whole-cell patch-clamp recordings. Consistent with the elevation in MFR from MEA recordings, Yoda1 exposure resulted in an increase in the frequency of calcium transients. As expected for a cation-permeable channel, Yoda1 administration produced a shift in the reversal potential of cortical neuron cell membranes under voltage clamp conditions. Our work suggests that cortical neurons express functional PIEZO1 channels and demonstrates the electrophysiological effects of channel activation in vitro.

## 2. Materials and Methods

### 2.1. Isolation, Cell Culture, and Device Preparation

To create the embryonic-derived cortical cultures, 5 pregnant mice (4 weeks old; Envigo RMS Inc., Indianapolis, IN, USA) were used, where each dam yielded an average of 10 embryos. All surgical procedures were performed in accordance with the University of Texas at Dallas’s Institutional Animal Care and Use Committee. Primary derived cortical cells were cultured on 48-well MEAs (M768-tMEA-48W, Axion Biosystems, Atlanta, GA, USA) for electrophysiological recording, 24-well culture plates (Corning, Durham, NC, USA) with RD German coverslips at the bottom (Thermo Fisher Scientific, Waltham, MA, USA) for immunocytochemical (ICC) characterization, and 38 mm polystyrene plates (Corning, Durham, NC, USA) for patch-clamp and calcium imaging studies.

The day prior to seeding, the MEA plate and calcium imaging plates were coated with 0.1 mg/mL Gibco^TM^ Poly-D-Lysine (PDL) (Thermo Fisher Scientific, Waltham, MA, USA), and plates used for patch-clamp were coated with 0.2 mg/mL PDL and laminin (Sigma-Aldrich, St. Louis, MO, USA). All cells were incubated at 37 °C with 95% air/5% CO_2_ overnight. On the seeding day, PDL was washed three times with deionized (DI) water, then devices were coated with 20 µg/mL Laminin (Sigma-Aldrich, St. Louis, MO, USA), and incubated at 37 °C and 95% air/5% CO_2_ for at least two hours.

Embryos were removed by making a V-shaped cut on the lower abdomen of the anesthetized pregnant female mice and were immediately transferred into ice-cold Leibovitz’s L-15 Medium (Thermo Fisher Scientific, Waltham, MA, USA). Individual embryos were removed from the amniotic sacs, decapitated, and stored in ice-cold L-15. Frontal lobes were dissected in a trapezoidal pattern, and the tissue was transferred to a falcon tube with BrainBits Hibernate Eb Complete Medium (Thermo Fisher Scientific, Waltham, MA, USA). Then, the Hibernate medium was removed and replaced with an enzyme buffer composed of Worthington Biochemical Corporation PDS (Papain) and Worthington Biochemical Corporation Hepatocyte Isolation (DNase) (Thermo Fisher Scientific, Waltham, MA, USA) reconstituted in DMEM+ media composed of Gibco^TM^ DMEM+ Glutamax (Thermo Fisher Scientific, Waltham, MA, USA) supplemented with 2% Gibco^TM^ B27 Supplement (50X) (Thermo Fisher Scientific, Waltham, MA, USA) and 5% Gibco^TM^ Horse Serum (Thermo Fisher Scientific, Waltham, MA, USA), 0.2% L-Ascorbic Acid (Sigma-Aldrich, MO, USA), and 1% Cytiva HyClone^TM^ Antibiotic/Antimycotic Solution (Thermo Fisher Scientific, Waltham, MA, USA) and incubated for 15 min at 37 °C and 95% air/5% CO_2_. Following dissociation, the tissue was mechanically triturated, and warmed DMEM 5/5 (DMEM+ media supplemented with 5% Fetal Bovine Serum (Thermo Fisher Scientific, Waltham, MA, USA) was added to the tissue and centrifuged at 2500 rpm for 5 min. Lastly, the supernatant was removed and cells were resuspended in warmed DMEM 5/5.

Cells were incubated for 4 h to enable adhesion. Following adhesion, DMEM 5/5 was added to all dishes. Cells were fed with DMEM+ every other day for 21 days. To achieve a neuron-enriched culture for calcium imaging study, we removed the horse serum from the DMEM+ media and performed a 1:1000 dilution of 5-Fluoro-2′-deoxyuridine and Uridine (FrdU) in the media.

### 2.2. Immunocytochemistry

After maturation to day in vitro (DIV) 21, cells cultured in a 24-well culture plate were fixed with 4% Paraformaldehyde (Sigma-Aldrich, St. Louis, MO, USA) in sterile Phosphate-Buffered Solution (PBS) for 10 min at room temperature. Afterwards, cells were washed three times for 5 min each with ice-cold PBS and were stored in PBS containing 0.1% sodium azide at 4 °C overnight. The next day, cells were permeabilized by incubating the samples for 10 min in PBS containing 0.3% Triton^TM^ X-100 (Sigma-Aldrich, St. Louis, MO, USA) and 10% Invitrogen Normal Goat Serum (NGS) (Thermo Fisher Scientific, Waltham, MA, USA). After washing the cells three times with PBS, cells were incubated in a blocking buffer containing 10% NGS in PBS for 2 h. Next, the blocking solution was removed, primary antibodies were introduced, and cells were stored at 4 °C overnight. The following primary antibodies were used: Abcam Chicken Anti-GFAP (1:700), Abcam Mouse Anti-NeuN (1:700), and Novus Biologicals Rabbit Anti-PIEZO1 (1:200). The following day, cells were washed three times with PBS and incubated with secondary antibodies in PBS containing 10% NGS for 1 h at room temperature in the dark. The secondary antibodies were Abcam Goat Anti-Chicken IgY H&L (Alexa Fluor647, 1:1000), Goat Anti-Mouse IgG H&L (Alexa Fluor 555, 1:1000), and Goat Anti-Rabbit IgG H&L (Alexa Fluor 488, 1:1000). All primary and secondary antibodies were purchased from Thermo Fisher Scientific. Then, wells were treated with 1× EverBrite Trueblack Hardest Mounting Medium (VWR, Radnor, PA, USA) for 30 s and washed three times. Finally, glass coverslips were mounted on top of the cover slides by applying Fluoromount^TM^ Aqueous Mounting Medium (Sigma-Aldrich, St. Louis, MO, USA) onto the slides. The edges were sealed with clear nail polish.

Confocal imaging was performed at 40× magnification (Eclipse Ti, Nikon, Tokyo, Japan). Regions of interest (ROI) were acquired to determine the percentage of NeuN-positive cells in the existing culture. Each ROI was then processed in ImageJ (NIH)to quantify the expression of PIEZO1 in the neurons. Briefly, a color threshold was applied to the single and merged channels, and the number of cells was counted manually. We opted for manual counting due to the nature of cortical neuronal networks. The high density of cells posed challenges for accurate individual cell counting using automated approaches such as ImageJ. A similar process was performed to quantify the percentage of GFAP-positive cells and PIEZO1-positive astrocytes.

For image analysis, we performed a two-sample *t*-test using Origin (OriginLab Corporation, Northampton, MA, USA) to determine if the expression of PIEZO1 is significantly different in one of the cell types. The results were considered statistically significant if the *p*-value was less than 0.05.

### 2.3. Extracellular Recording Using Microelectrode Arrays

Electrophysiology recordings were acquired at DIV22 from five separate 48-well MEA plates on five different days with a Maestro Classic or the Maestro Pro system (Axion Biosystems, Atlanta, GA, USA) at a 12.5 kHz sampling rate. The characteristics of the MEA plate are provided in Table 1. We focused the study on stable cultures that had reached at least DIV21, a time point when cortical networks typically undergo maturation and establish functional connectivity [23,31].

The recordings were conducted in a humidified 95% air/5% CO_2_ environment and maintained at 37 °C via the integrated heating plate. Signals were band-pass filtered (single pole Butterworth, 300–5000 Hz), and individual action potentials were detected with an adaptive threshold of ±5.5 σRMS per electrode in AxIS data acquisition software (Axion Biosystems, Atlanta, GA, USA). After transferring the MEA plate to the recording environment, we allowed the cultures to habituate to the new environment for at least 10 min and then recorded a minimum of 5 min of baseline spontaneous activity. 

### 2.4. Pharmacological Preparation and Exposure

#### 2.4.1. Pharmacological Exposure and Analysis Using Microelectrode Arrays

For the introduction of pharmacological agents, a stock solution of the mechanosensitive channel blocker, GsMTx4 (Tocris Bioscience, Thermo Fisher Scientific, Waltham, MA, USA), was prepared. To prepare the GsMTx4 stock solution, we added up to 4 µL of a concentrated stock into the MEA wells. This was carried out to avoid dilution effects from the recording media. Since we aimed for a concentration of 3 µM GsMTx4 per well, and considering each well has 400 µL of medium, we prepared a stock solution with a concentration of 244 µM. In addition, a stock solution of Yoda1 (Tocris Bioscience, Thermo Fisher Scientific, Waltham, MA, USA) was prepared by dissolving Yoda1 into DMSO for a final concentration of 20 mM. The stock solution was diluted in DMEM+ to obtain six different desired concentrations for vehicle experiments.

**Figure 1:**
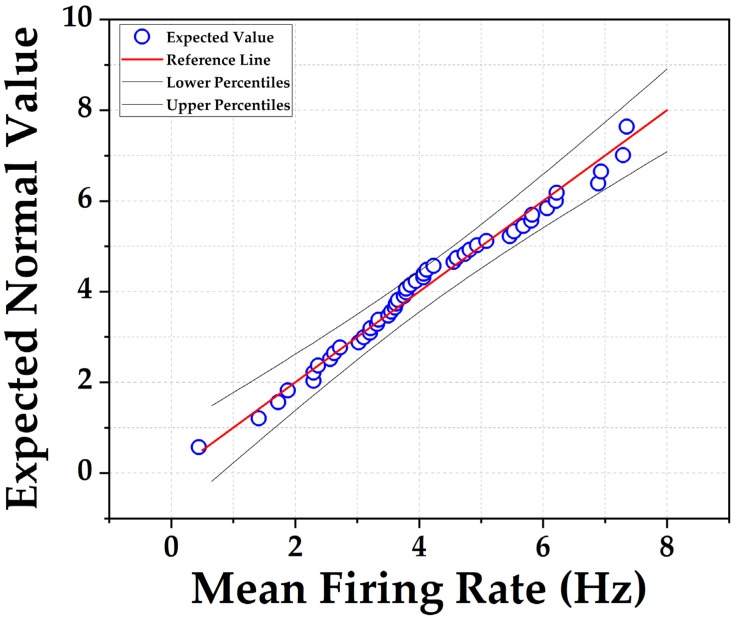
Quantile–quantile (QQ) plot depicts the normal distribution of mean firing rate (MFR) of 48 individual neuronal networks, each containing approximately 105 cells. MFR values were obtained from individual networks on a typical MEA plate under control conditions.

We determined the change in mean firing rate (MFR) from electrode sites with identifiable spike activity after introducing Yoda1 and GsMTx4 to the separate wells. Additionally, we investigated the inhibitory effect of GsMTx4 on Yoda1-induced spike activity. The maximum concentration of DMSO was 0.5% and in control experiments; we verified that 0.5% DMSO alone had no effect on any of the measurements. Each experiment typically lasted between 1 to 2 h. To process the MFR for each well, NeuralMetrics software (Axion Biosystems, Atlanta, GA, USA) was used. For statistical analysis of mean firing rates, it is well documented that inter-spike intervals from individual neurons exhibit a skewed distribution [32]. However, quantifying the MFR for each network within a recording well consists of the sum of activity derived from 16 electrodes in each well, providing a more comprehensive picture of network activity. Consistent with the central limit theorem, the distribution of MFR values for multiple networks is well approximated by a normal distribution, as shown in Figure 1. Statistical tests that assume normality were considered appropriate for data analyses. Using Origin, a paired *t*-test was performed for statistical analysis comparing MFR before and after treatment for individual treatment groups where *p* < 0.05 was statistically significant. Plots were also generated via Origin.

The ability of MEA to resolve action potentials extracellularly can be influenced by various technical specifications, such as electrode diameter/impedance, density, and spacing. It is important to note that recorded electrical signals primarily capture high-frequency components known as multiunit activity. The resolution of single units typically requires additional techniques, such as spike sorting. Therefore, we used a highly conventional method to identify single units for both in vivo and in vitro neural recordings [33,34,35]. First, we converted the AxIS spike (.spk) files to .nex files using Axion Data Export Tool (Axion Biosystems, Atlanta, GA, USA). Then, we imported the .nex files into Offline Sorter (Plexon Inc., Dallas, TX, USA), and spikes were then manually sorted into characteristic waveforms (single units) based on separation in two-dimensional (2D) principal component (PCA) space using Offline Sorter software. The 2D PCA space showing the recorded spikes was then sorted for each channel into well-resolved units using the Scanning K-means method in Offline Sorter and results were confirmed by visual inspections. The inclusion criteria for subsequent analyses required that: (1) the unit consisted of at least 30 spikes acquired during a 4 min recording period (2 min before and 2 min after pharmacological treatment); and (2) a peak-to-peak single unit amplitude of at least 20 µV. 

#### 2.4.2. Calcium Imaging

Calcium imaging was performed as previously described by Li and colleagues [36]. Following the 21 days of culture maturation, cells were incubated with 4 μM calbryte 520-AM at 37 °C for 30 min. After the incubation, cells were washed with bathing solution comprising the following (in mM): 13.5 NaCl, 5 KCl, 10 HEPES, 2 CaCl_2_, 1 MgCl_2_, 10 glucose, adjusted to pH 7.4 with osmolality of 295–305 mOsm/L. A custom-made perfusion system was used to deliver 30 μM Yoda1, diluted from concentrated stock solution into the bathing solution, to the cortical neuron cultures at a flow rate of 500 µL per second. While perfusing, calcium dynamics images were recorded using an Olympus IX73 inverted microscope (40× magnification, FITC, 100 ms exposure time). 

A total of six cell culture plates were used, each containing neuron-enriched cortical culture. Within each plate, a minimum of six neurons were selected within individual field of view. Calcium transients were recorded during the baseline and following Yoda1 administration. For data analysis, the intensity values of calcium transients for all six neurons within the field of view were averaged and the change in fluorescence intensity with respect to the baseline fluorescence intensity (ΔF/F) was calculated. Statistical analysis relied on paired-sample Wilcoxon rank tests to compare peak intensity values between baseline and post-treatment conditions, as well as to assess signal frequency variations in both states. 

#### 2.4.3. Whole-Cell Patch Clamp Electrophysiology

Embryonic-derived cortical neurons were cultured for patch-clamp electrophysiology as described previously and seeded on 38 mm polystyrene plates (Corning, Durham, NC, USA) coated with 0.2 mg/mL PDL and laminin (Sigma-Aldrich, St. Louis, MO, USA). Cells were kept in a 37 °C, 95%/5% CO_2_ incubator for 21 days, and fed with DMEM+ media every other day. Whole-cell patch-clamp electrophysiology was conducted using a Multiclamp 700B (Molecular Devices, San Jose, CA, USA) amplifier and pCLAMP 9 acquisition software (Molecular Devices, San Jose, CA, USA). Recordings were sampled at 10 kHz and filtered at 1 kHz (Digidata 1550B, Molecular Devices). Micropipettes (outer diameter 1.5 mm: inner diameter, 0.89 mm; BF150-110-10, Sutter Instruments) were pulled using a PC-100 puller (Narishige, Amityville, NY, USA) and fire polished to a resistance of 3–5 MΩ using a microforge (MF-83, Narishige). Series resistance was typically 7 mV and was compensated up to 70%. Data were analyzed using Clampfit 10 (Molecular Devices, San Jose, CA, USA).

Voltage clamp recordings were performed to assess the whole-cell current in each of the experimental conditions. Recordings were taken using a pipette solution containing (in mM): 135 KCl, 10 HEPES, 4 ATP-Mg, 0.9 GTP-Na, 0.5 EGTA, and 5 NaCl adjusted to a pH of 7.4 with an osmolarity ranging from 305–320. The external bath solution contained (in mM): 135 NaCl, 5 KCl, 2 CaCl_2_, 1 MgCl_2_, 20 mM sucrose, 10 glucose, and 10 HEPES, with a pH of 7.4 and osmolarity ranging from 310–320 mOsm/L [37]. The cell capacitance, whole-cell compensation, fast and slow capacitance, resting membrane potential, leak current, pipette resistance, and offset were recorded for each cell.

Once a giga-ohm seal was achieved on a cell, small bursts of suction were used to rupture the patch on the cell, followed by application of compensation for fast and slow capacitance, before applying whole-cell mode on the Multiclamp. Perfusion of standard bath solution was initiated, and 3 voltage clamp measurements were taken. A standard voltage clamp recording was conducted by holding the cell at −70 mV, stepping it to 0 mV, and returning to a holding potential of −70 mV to assess the health of the cell and the quality of the patch. Next, a voltage step protocol was applied that steps the cell from −100 mV to +70 mV [38]. Clamped cells were then held at −70 mV while bath perfusion continued for 30 s, after which perfusion was switched to bath solution containing 30 µM Yoda1. Following Yoda1 application to the cells for 2 min, the voltage clamp protocols were repeated under conditions of continuous flow of bath containing Yoda1.

## 3. Results

### 3.1. PIEZO1 Channels Are Expressed in Murine Frontal Cortical Neurons

**Figure 2:**
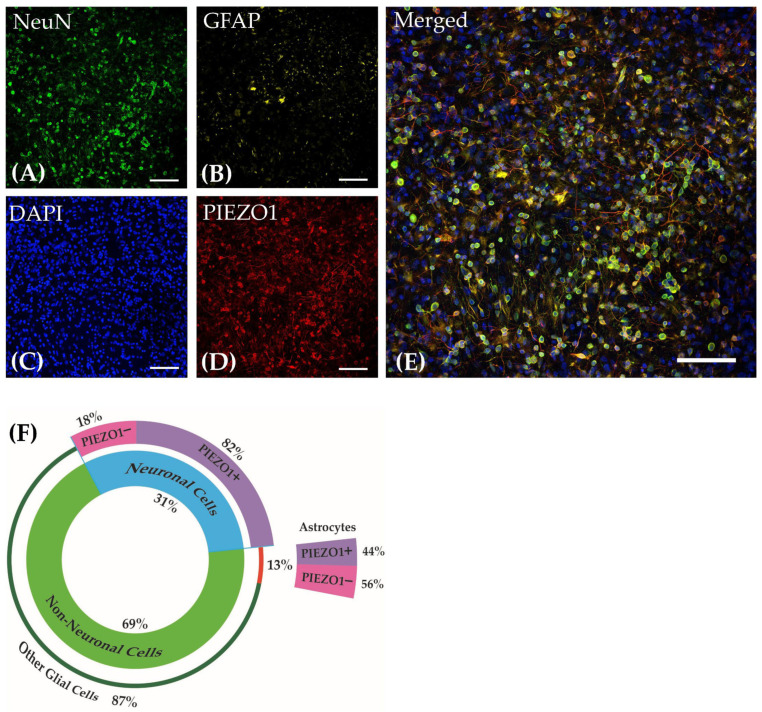
Cortical cultures derived from embryonic mice on DIV21 stained for (**A**) Neuronal Nuclear Protein (NeuN), (**B**) Glial Fibrillary Acidic Protein (GFAP), (**C**) DAPI, and (**D**) PIEZO1. (**E**) Merged image of all channels. Images were taken at 40× magnification. Scale bar = 50 µm. (**F**) The pie chart reflects the percentage distribution of the cell types in the cortical culture and their co-localization percentage with PIEZO1.

Cultured cells derived from embryonic tissue form dense networks of neurons and astrocytes on adherent surfaces. As shown in Figure 2A–D, cultures at DIV21 were labeled for NeuN, GFAP, PIEZO1, and DAPI. The merged image is shown in Figure 2E. The analysis of three distinct regions of interest (ROIs) captured from three separate cultured wells demonstrated that the culture comprised an average of 31.0 ± 0.1% neurons and 69.0 ± 0.1% (mean ± SEM) non-neuronal cells. Approximately 13.0 ± 0.1% (mean ± SEM) were identified as astrocytes among the non-neuronal cells. Each ROI contained a minimum of 500 cell bodies that were stained with DAPI. Quantifying the same ROIs with at least 150 neurons in each ROI showed that 82.0 ± 0.1% (mean ± SEM) of the neurons identified by NeuN labeling co-express PIEZO1. In contrast, 44 ± 0.2% of astrocytes identified by GFAP labeling co-express PIEZO1 where at least 60 astrocytes were counted in each ROI. These observations suggest that PIEZO1 is predominately expressed in the neuronal population within these cultured cortical networks. The distribution of cellular expression is visually summarized in the pie chart presented in Figure 2F.

### 3.2. Cortical Neuronal Networks Activity under Control Conditions

**Figure 3:**
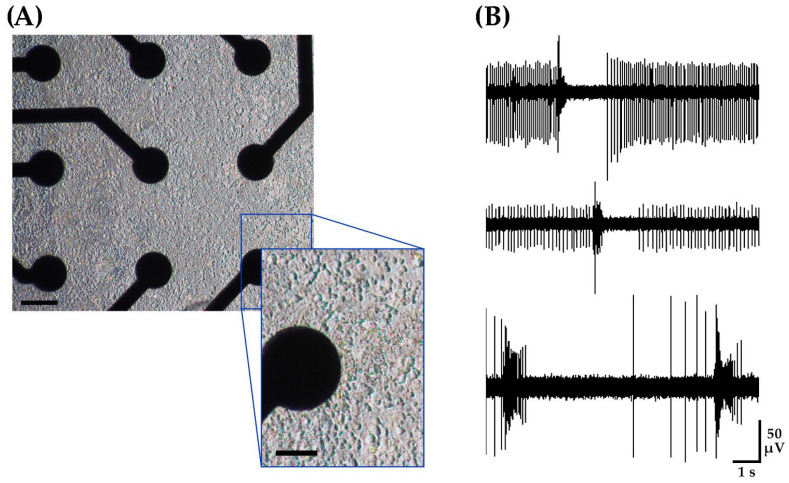
Cortical neuronal networks recording under control conditions using a 48-well microelectrode array. Each network contains approximately 105 cells. (**A**) DIV21 mouse embryonic-derived cortical networks. Scale bar = 50 µm. Inset scale bar = 25 µm. (**B**) Typical recording from three representative channels under control conditions demonstrating single spikes and bursts.

For each MEA plate, an examination of 768 electrode sites from 48 networks derived from four embryos from a mouse dam showed an active electrode yield of 99%, where at least one unit could be distinguished on each electrode site (Figure 3A). Here, a unit refers to a class of consistent waveforms that can be grouped based on similar profiles. This grouping is often considered to be the product of a distinct neuron. Unit activity refers to the expression of spike potentials either occurring in isolation or as a burst, which is a train of spikes. For a detailed analysis of single units, a subset of high signal-to-noise recordings was selected from four separate wells of the MEA, which consisted of 1.7% of the overall dataset. For this subset, the average peak-to-peak amplitude of detected activity ranged from 49 to 155 µV averaging to 92 ± 8 µV (mean ± SEM, *n* = 13 electrodes derived from four wells). Of this subset of recordings, all channels demonstrated multiple units with an overall firing rate of 8.15 ± 2 Hz (mean ± SEM, *n* = 13 electrodes). Figure 3B shows the raw voltage trace of three representative channels.

Furthermore, the analysis of three untreated wells of the MEA, each with a minimum of 50% active electrodes, did not reveal any time-dependent alteration in spontaneous activity, indicating stability of the measurements. Comparing the 5 min baseline period with an MFR of 2.2 ± 0.3 Hz (mean ± SEM) to the last 5 min period of a 2 h recording with an MFR of 1.9 ± 0.3 Hz, there was no significant difference observed at a significance level of *p* < 0.05. Overall, our observations with the multi-well MEA system were consistent with prior work, which showed unit activity in the form of single spikes and bursts [26,39], where coordinated bursting was readily apparent.

### 3.3. Pharmacological Modulation of Cortical Neuronal Networks

#### 3.3.1. PIEZO1 Agonist, Yoda1, Transiently Increases the Neuronal Networks MFR

**Figure 4:**
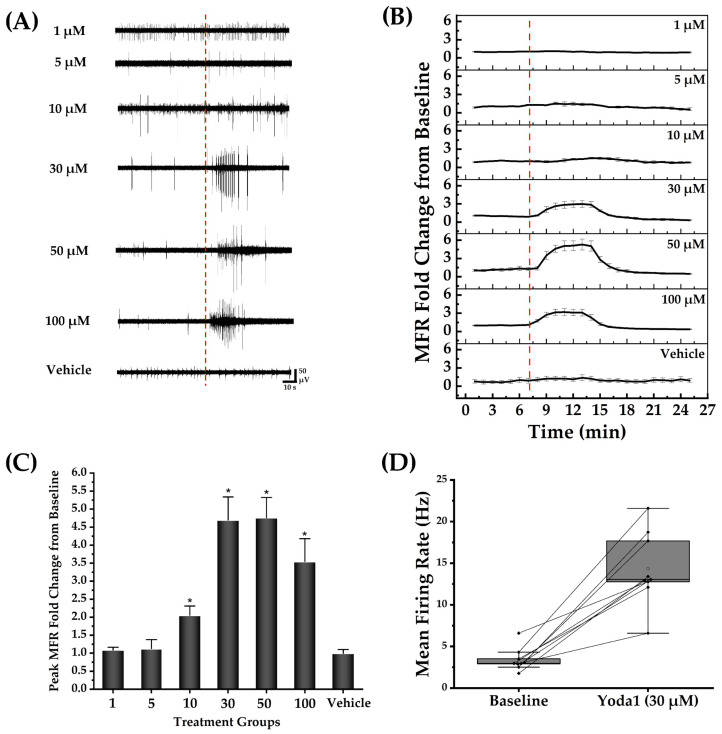
Yoda1 transiently enhanced the mean firing rate of the neuronal networks. A total of 10^5^ viable cells were seeded per cultured network. The number of cultured networks for each concentration represented in (**B**,**C**) is as follows: 1, 5, 50, and 100 µM (*n* = 4 networks); 10 µM (*n* = 10 networks); 30 µM (*n* = 9 networks); and vehicle (DMSO control, *n* = 3 networks). (**A**) Representative electrode raw voltage traces showing the transient elevation of spike activity after exposure to different concentrations of Yoda1 but not the largest concentration of vehicle (DMSO control). The red dotted line indicates the Yoda1 and DMSO (vehicle) administration time points. (**B**) Normalized MFR versus time for the Yoda1 treatment groups and control. The time corresponding to Yoda1 and DMSO (vehicle) administration is represented by the red dotted line. (**C**) Peak fold change in MFR from the baseline for each of the Yoda1 concentrations and vehicle. The results of the paired *t*-test showed a significant increase in MFR from the baseline in the groups treated at 10, 30, 50, and 100 µM of Yoda1. No significant change was identified in the lower concentrations of Yoda1 and the largest concentration of DMSO (vehicle). * indicates a statistically significant difference at *p* < 0.05. (**D**) Box plot illustrating the effect of 30 µM of Yoda1 on activity of nine representative neuronal networks.

Following a 5 min baseline recording of spontaneous activity at DIV22, cortical networks were treated with Yoda1 or vehicle alone, which consisted of the largest concentration of DMSO used as a solvent for the agonist. Figure 4A shows recordings of unit activity in representative channels before and immediately following administration of Yoda1, revealing a transient increase in unit activity. After normalization to the baseline firing rate for each well, time course data were summarized as fold changes in MFR as a function of the concentration of Yoda1 (Figure 4B, mean ± SEM, *n* = 4 networks at 1, 5, 50, and 100 µM, *n* = 10 networks at 10 µM, *n* = 9 networks at 30 µM, *n* = 3 for vehicle). For Yoda1 concentrations of 10, 30, 50, and 100 µM, the increase in the MFR typically lasted between 30–50 s. Figure 4C summarizes the transient elevation of MFR for each concentration tested. A box plot with connected lines in Figure 4D illustrates the effect of 30 µM Yoda1 administration where changes were quantified in terms of MFR rather than normalized to baseline values for each network. Likewise, Table 2 presents the unnormalized MFR values for the baseline and post-exposure to different concentrations of Yoda1 and 0.5% DMSO. Our analysis demonstrated that regardless of whether the data were normalized or not, the interpretation regarding the effect of transient elevation in MFR induced by Yoda1 on the cortical neuronal networks remained the same. We also observed a decrease in MFR from the baseline in the same treatment groups at 30, 50, and 100 µM Yoda1 following the transient increase in MFR. This decrease in MFR lasted for 20–40 min across the three concentrations and was followed by a slow recovery to the baseline MFR for each cortical network. In addition, the burst rate data revealed a significant increase (*p* < 0.05) following exposure to Yoda1. The baseline burst rate measured 0.09 ± 0.01 Hz, while the post-exposure burst rate was measured 0.30 ± 0.02 Hz.

#### 3.3.2. GsMTx4 Inhibits Spontaneous and Yoda1-Induced Activity in Cortical Neuronal Networks

**Figure 5:**
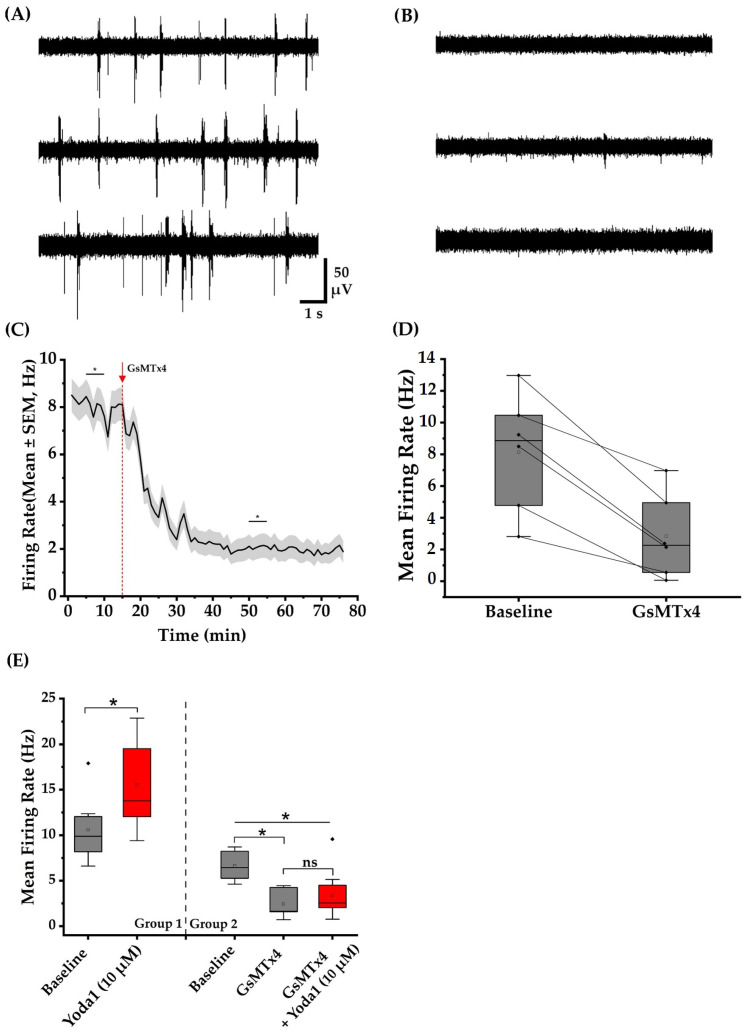
GsMTx4 suppressed spontaneous and Yoda1-induced cortical neuronal networks activity. Each network contains approximately 10^5^ viable cells. (**A**) Representative voltage traces from three channels under baseline conditions. (**B**) Voltage trace from the same three representative channels 35 min after exposure to GsMTx4. (**C**) The line graph shows time-dependent decrease in the MFR after the administration of GsMTx4. The arrow and the dotted red line indicate the time point when GsMTx4 was administered. The asterisks indicate a statistically significant difference at a level of *p* < 0.05 when comparing this time range across multiple wells. (**D**) Six representative neuronal networks, depicted by diamonds, showing a decrease in MFR after the administration of GsMTx4. The squares represent the mean value of the data. (**E**) Box plots demonstrate a significant increase in MFR following the administration of Yoda1 to 10 MEA wells. Pretreatment of 10 separate wells with GsMTx4 effectively inhibited the Yoda1-induced activity increase at 10 µM. * indicates a statistically significant difference at *p* < 0.05. Diamonds depict the outliers, empty squares represent the mean value, and ‘ns’ indicates non-significance *(p* > 0.05).

Following a 15 min baseline recording of spontaneous activity at DIV22, we added GsMTx4 stock solution to 12 separate wells to achieve a final concentration of 3 µM GsMTx4 in each well. Interestingly, in the absence of any exogenous mechanical stimuli, the recordings showed a decrease in MFR of the neuronal networks with exposure to GsMTx4 (Figure 5C). A paired-sample *t*-test revealed a statistically significant decrease (*p* < 0.05) in MFR when comparing a 5 min baseline to a 5 min post-treatment period with GsMTx4 during which the MFR reached a steady state level. Furthermore, we observed a decrease in the bursting properties of neurons following exposure to the toxin. After exposure to GsMTx4, the burst rate and burst duration fell significantly by 92.0 ± 0.1% (*p* < 0.05) and 33.0 ± 0.1% (*p* < 0.05), respectively. Table 3 summarizes all the values associated with baseline and post-exposure to GsMTx4 conditions. Our observations suggest that the activity of a mechanosensitive ion channel contributes to the baseline spike firing of cultured neuronal networks. GsMTx4 is known to target mechanosensitive ion channels, including PIEZO1 and the transient receptor potential canonical 1 (TRPC1) [27,40].

Next, we examined whether adding GsMTx4 inhibits the transient elevation in MFR induced by Yoda1 at different concentrations. GsMTx4 was introduced into 19 separate wells at a final concentration of 3 µM. After a 10 min incubation period, 10 µM Yoda1 was introduced into 10 wells pre-treated with GsMTx4, while 30 µM Yoda1 was added to the remaining 9 wells. An ANOVA test revealed that 3 µM of GsMTx4 significantly decreased the MFR of neuronal networks and inhibited the Yoda1-induced increase in MFR at 10 µM (*p* < 0.05). Furthermore, statistical analysis revealed a significant difference (*p* < 0.05) between Yoda1-treated wells (Group 1) and those pre-treated with GsMTx4 followed by Yoda1 exposure (Group 2) (Figure 5E). No inhibitory effect of GsMTx4 was observed for 30 µM Yoda1.

#### 3.3.3. Increase in Peak-to-Peak Amplitude of the Spikes with Exposure to Yoda1

**Figure 6:**
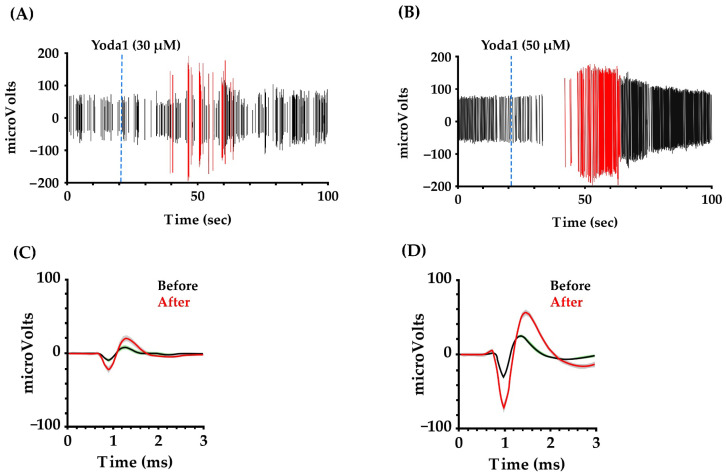
Yoda1 exposure shifted the peak-to-peak amplitude of the spikes. (**A**,**B**) show 100 s of recording from representative units on networks treated with Yoda1 at 30 and 50 µM concentrations, respectively. The time corresponding to Yoda1 administration is represented by the blue dotted line. Spikes with lower peak-to-peak amplitude are depicted by black lines, whilespikes with higher peak-to-peak amplitude are represented by the red lines. (**C**,**D**) show unit waveforms (mean ± STD) detected before and immediately after administration of Yoda1 at 30 and 50 µM, respectively.

We performed unit sorting on Yoda1-treated networks at 30 and 50 µM to document any effect of Yoda1 on spike amplitude. We focused on 10 representative electrode sites from both concentrations with the highest signal-to-noise baseline recording to facilitate analysis. In the networks treated with 30 µM concentration of Yoda1, we focused on at least 34 units recorded by 10 different electrode sites. Among these single units, 13 units (38%) showed a shift in peak-to-peak amplitude after Yoda1 administration. For 50 µM Yoda1, 15 of 31 detected units (48%) showed an increase in ensemble unit peak-to-peak amplitude, as shown in Figure 6. These results suggest that the agonist produces a transient change in the total sink-source current that underlies the basis of extracellular action potentials [41].

#### 3.3.4. Yoda1 Increases the Calcium Transient Frequency in Cortical Neurons

**Figure 7:**
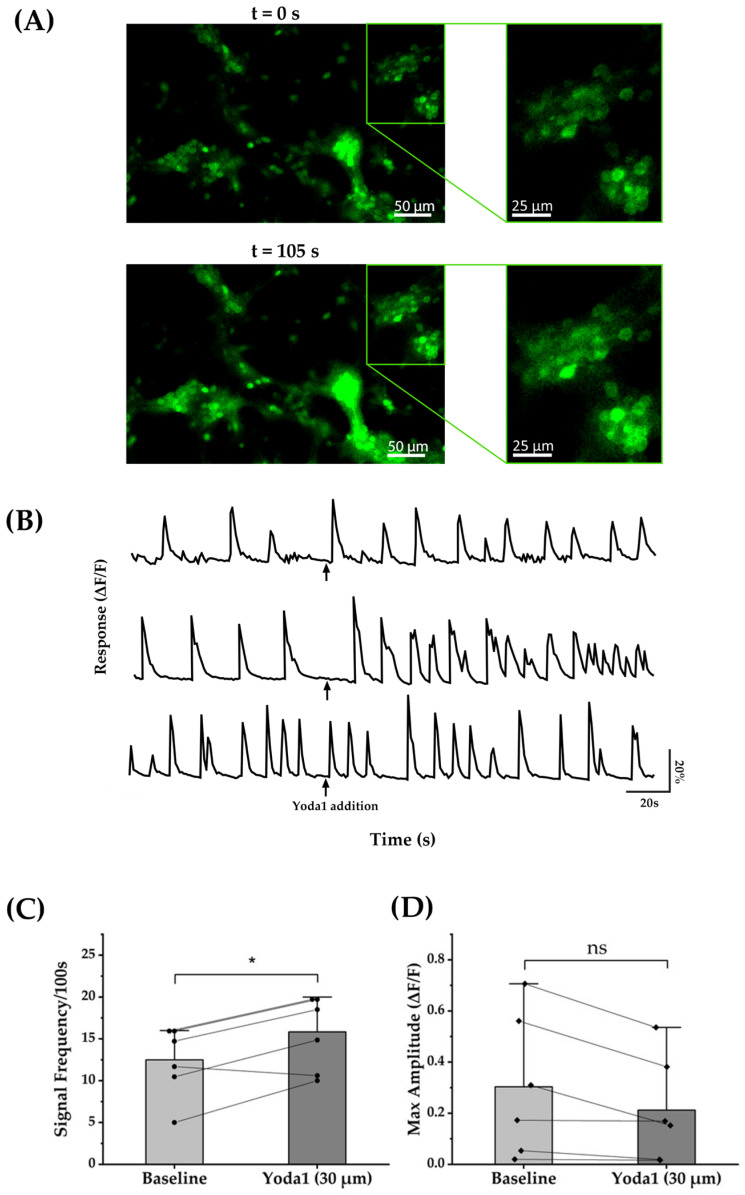
Yoda1 increased the frequency of calcium transient in cortical cells. (**A**) Fluorescence intensity images representing the intracellular calcium concentration in neuronal cells before (*t* = 0 s) and after (*t* = 105 s) application of Yoda1. (**B**) Time-dependent average fluorescence intensity relative to the baseline (ΔF/F) for three representative wells showing an increase in the rate of spontaneous calcium transients with exposure to Yoda1. (**C**) Comparison of the rate of calcium transients between the 100 s baseline and 100 s post-exposure to Yoda1 demonstrates a statistically significant increase at a level of *p* < 0.05. Circles depict values from six separate wells. (**D**) The peak amplitude of the calcium transients 100 s before and 100 s after Yoda1 treatment did not show any significant difference (ns). Analysis was conducted on six separate wells, each containing approximately 25 × 10^4^ viable cells. Each diamond represents the value obtained from one of the six wells. * indicates a statistically significant difference at *p* < 0.05.

To complement the MEA results, we performed calcium imaging on neuron-enriched cultures where ROIs consisting of neurons could readily be identified. Fluorescence intensity recordings corresponding to the intracellular calcium concentration were recorded 100 s before and after exposure to the Yoda1. Representative images in Figure 7A show the change in fluorescence intensities following Yoda1 treatment. As shown by the representative fluorescence intensity recordings in Figure 7B, administration of 30 µM Yoda1 resulted in a marked increase in the frequency of calcium transients in cortical neurons. Quantitative analysis across multiple ROIs showed a statistically significant increase of 25.6% in the rate of spontaneous calcium transients (Figure 7C). Yoda1 exposure did not appear to affect the maximum amplitude of the calcium transients (Figure 7D). Overall, the observation of a rise in the rate of calcium transients with Yoda1 is consistent with the MEA findings.

#### 3.3.5. Yoda1 Alters the Reversal Potential of Cortical Neurons

**Figure 8:**
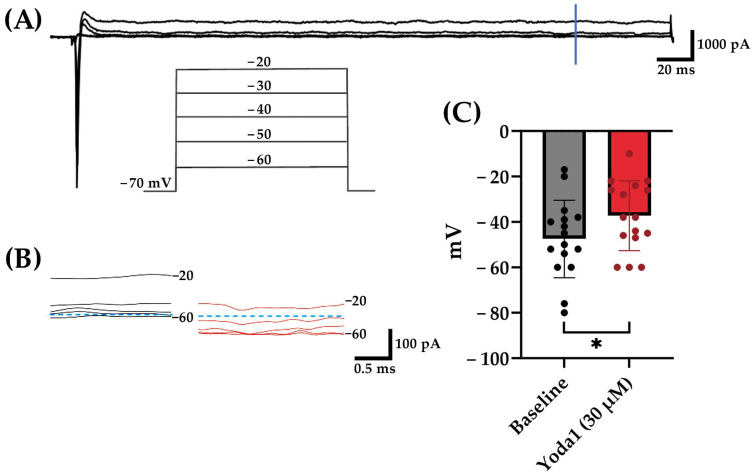
Yoda1 exposure induced a statistically significant shift in reversal potential. (**A**) Representative membrane current traces, evoked by the voltage step protocol shown in the inset during application of bath solution, with a vertical blue line indicating where quantitative measurements were performed. (**B**) Representative current traces at 300 ms before (black) and after (red) 30 µM Yoda1 application at voltages from −60 to −20 mV. Blue dashed lines denote 0 pA levels. (**C**) Statistically significant shift in cellular reversal potential from baseline with exposure to 30 µM Yoda1 (*p* = 0.015). * = *p* < 0.05. Analysis was conducted on 16 recorded neurons.

To assess the effect of Yoda1 on individual cortical neurons, voltage clamp experiments were conducted to measure whole-cell current in the cells. Cells were voltage-clamped at −70 mV and stepped from −100 mV to +70 mV in increments of 10 mV to record evoked membrane currents. As expected, depolarizations induced a transient inward current consistent with voltage-gated Na^+^ channel behavior followed by outward currents (Figure 8A). To quantify the effects of Yoda1, membrane current magnitudes were quantified at 300 ms after the initiation of the voltage steps to allow for Na^+^ channel inactivation and K^+^ channel steady-state. The reversal potential under each condition was determined based on the voltage where the membrane currents were zero. Yoda1 exposure resulted in a statistically significant shift in reversal potential (Figure 8C). This observation is consistent with the activation of a non-specific cationic channel by the PIEZO1 agonist Yoda1.

## 4. Discussion

In this study, we investigated the expression and effect of pharmacological modulation of PIEZO1 channels on the mouse embryonic-derived cortical networks. The use of murine neuronal networks cultured on substrate-integrated microelectrode arrays constitutes a common platform that has been the basis of a wide range of studies spanning neural dynamics/computation [42,43,44], biosensing [45], and neuropharmacology [20,31,46]. Our work is among the first to characterize the contribution of mechanosensitive ion channel activity to the spontaneous firing of cortical networks. Using immunocytochemistry, we first demonstrated that PIEZO1 channels are expressed in neurons and astrocytes. Exposure to the mechanosensitive ion channel inhibitor, GsMTx4, demonstrated a decrease in the MFR of the cultured neuronal networks in MEA plates, whereas the PIEZO1 channel agonist, Yoda1, transiently elevated the MFR and burst rate of the neuronal networks. Furthermore, we demonstrated that Yoda1 exposure, in a data subset consisting of isolated units, appears to induce a transient increase in the peak-to-peak amplitude of the spikes. To complement the MEA findings, we conducted intracellular calcium and whole-cell voltage clamp measurements showing that Yoda1 increases the rate of spontaneous calcium transients and produces a depolarizing shift in the cellular reversal potential, respectively. The calcium transient results are largely consistent with the present MEA findings as well as prior work [29,47]. With respect to the whole-cell voltage clamp work, because we measured total current without any channel blockers, the change in reversal potential to more positive potentials is consistent with the Yoda1-induced activation of a cation current with a positive equilibrium potential, aligning with literature reports of PIEZO1 kinetics [48]. Taken together, these data suggest that PIEZO1 is expressed in cortical neurons and is capable of contributing to increased excitability of these networks.

To date, relatively few studies have reported the presence and role of PIEZO1 channels in rodent cortex. In cortical astrocytes, PIEZO1 upregulation may act to dampen neuroinflammation [14]. Work by Velasco-Estevez and colleagues showed that PIEZO1 is expressed in neurons in the mouse frontal cortex and may have a role in demyelination [12]. Wang et al. reported PIEZO1 expression in the cerebral cortex of adult rats, with data suggesting a role for these channels in ischemia/reperfusion injury [49]. Furthermore, it has been reported that sparing nerve injury induces abnormal PIEZO1 expression in parvalbumin-expressing interneurons (PV-INs) in the anterior cingulate cortex, leading to intracellular calcium accumulation, microglial phagocytosis, and subsequent reduction in PV-Ins [10].

The two-dimensional stiff substrates used in this work, may influence the inherent excitability of the neuronal networks. Prior work by Zhang and colleagues suggests that stiffer substrates enhance neuronal network activity, an effect primarily attributed to voltage-sensitive calcium channels [50]. Cultured neurons, particularly growth cones, can produce mechanical tension [51], where mechanosensitive channels are implicated for growth cone guidance [52,53]. As a result, we cannot exclude the possibility that the extent that mechanosensitive channels impact neuronal network activity may be influenced by in vitro versus in vivo conditions.

Interestingly, the transient increase in neuronal activity we observed with the chemical PIEZO1 agonist, Yoda1, bears a resemblance to the reported effects of persistent low-frequency ultrasound on cultured hippocampal neurons cultured on microelectrode arrays. Saccher and colleagues reported that persistent exposure to focused ultrasound produced transient elevations in spontaneous network activity [54]. Calcium imaging studies offer a link between the effects of ultrasound stimulation and mechanosensitive channels, including but not limited to PIEZO1 channels [18,19]. The observed inactivation following the transient increase in mean firing rate aligns with the reported kinetics of PIEZO1 channels. A characteristic of PIEZO1 is that, following mechanical stimulation, currents significantly decay, persisting even with continued stimulation [5,55,56]. Therefore, prolonged exposure to Yoda1 may potentially lead to inactivation.

Lastly, we observed a transient shift in the peak-to-peak amplitude of the spikes with exposure to Yoda1, which suggests that in a subset of recordings, there was an increase in the net transmembrane current underlying the biophysics of extracellular potentials. Note that there is a well-understood relationship between the transmembrane current of the source neuron and the first derivative of the action potential [40,57]. Our observation of a transient increase in the peak-to-peak amplitude of the extracellular spike with Yoda1 suggests that activation of PIEZO1 could alter the rate of rise of action potentials in neurons responding to mechanical stimuli. Consistent with this notion, Boada et al. reported that peripheral somatosensory neurons exposed to mechanical forces showed an increase in the first derivative of the action potential [58].

Our data are consistent with the notion that functional PIEZO1 receptors mediate changes in neuronal activity. However, a limitation of the present study is that we cannot exclude a role for PIEZO1 receptor modulation of astrocytes within the cultured networks contributing to neuronal spike modulation. Prior work has demonstrated crosstalk between astrocytes and neurons in health and disease [59]. It is possible that PIEZO1 activation could trigger elevations in intracellular calcium in astrocytes to promote the secretion of gliotransmitters that are excitatory to neurons [60]. However, note that we were able to see the effects of Yoda1 in neuron-enriched cultures through calcium imaging experiments.

In summary, mechanosensitive ion channels, including PIEZO1 channels, are expressed and contribute to the spontaneous firing activity in mouse embryonic-derived cortical networks. Inhibition of mechanosensitive channel activity can decrease network firing rate, whereas the transient elevation in spike rate associated with the PIEZO1 agonist is similar to that which has been observed for focused ultrasound. Our findings add valuable insights into the role of PIEZO1 in cortical network dynamics and suggest potential implications for neurological modulation.

## Figures and Tables

**Table 1:** Cytoview 48-well microelectrode array (MEA) technical specifications.

Electrode/Well	Electrode Diameter	Nominal Impedance
16 electrodes in a 4 × 4 configuration	50 µm	8–12 KΩ measured at 41.5 kHz

**Table 2:** Baseline and post-exposure mean firing rate for different treatment groups. Data reported as mean ± SEM; * denotes statistical significance at *p* < 0.05.

Treatment Groups	Baseline MFR	+Yoda1 MFR
Yoda1 Concentration (µM)		
1	6.5 ± 0.4	6.8 ± 0.2
5	5.7 ± 1.0	6.0 ± 1.0
10	2.4 ± 0.3	4.6 ± 0.6 *
30	3.4 ± 0.5	14.3 ± 1.4 *
50	2.4 ± 1.0	16.4 ± 2.5 *
100	5.7 ± 1.0	15.0 ± 2.0 *
DMSO (0.5%)	3.3 ± 0.8	3.4 ± 0.5

**Table 3:** Neuronal network activity metrics comparing 5 min baseline recording with 5 min recording at steady state after exposure to GsMTx4. Data reported as mean ± SEM; * denotes statistical significance at *p* < 0.05.

	Baseline	Post-Exposure
MFR(Hz)	8.0 ± 0.1	2.0 ± 0.1 *
Burst Rate (Hz)	0.5 ± 0.1	0.05 ± 0.1 *
Burst Duration (s)	0.14 ± 0.1	0.09 ± 0.1 *

## Data Availability

The data presented in this study are available on request from the corresponding author. The data are not publicly available due to ethical restrictions.

## References

[1] Qin L., He T., Chen S., Yang D., Yi W., Cao H., Xiao G. (2021). Roles of Mechanosensitive Channel Piezo1/2 Proteins in Skeleton and Other Tissues. Bone Res..

[2] Haselwandter C.A., Guo Y.R., Fu Z., MacKinnon R. (2022). Quantitative Prediction and Measurement of Piezo’s Membrane Footprint. Proc. Natl. Acad. Sci. USA.

[3] Lin Y.C., Guo Y.R., Miyagi A., Levring J., MacKinnon R., Scheuring S. (2019). Force-Induced Conformational Changes in Piezo1. Nature.

[4] Pathak M.M., Nourse J.L., Tran T., Hwe J., Arulmoli J., Dai Trang T.L., Bernardis E., Flanagan L.A., Tombola F. (2014). Stretch-Activated Ion Channel Piezo1 Directs Lineage Choice in Human Neural Stem Cells. Proc. Natl. Acad. Sci. USA.

[5] Coste B., Mathur J., Schmidt M., Earley T.J., Ranade S., Petrus M.J., Dubin A.E., Patapoutian A. (2010). Piezo1 and Piezo2 Are Essential Components of Distinct Mechanically Activated Cation Channels. Science.

[6] Song Y., Li D., Farrelly O., Miles L., Li F., Kim S.E., Lo T.Y., Wang F., Li T., Thompson-Peer K.L. (2019). The Mechanosensitive Ion Channel Piezo Inhibits Axon Regeneration. Neuron.

[7] Tessier-Lavigne M., Goodman C.S. (1996). The Molecular Biology of Axon Guidance. Science.

[8] Liu T.-T., Du X.-F., Zhang B.-B., Zi H.-X., Yan Y., Yin J.-A., Hou H., Gu S.-Y., Chen Q., Du J.-L. (2020). Piezo1-Mediated Ca^2+^ Activities Regulate Brain Vascular Pathfinding During Development. Neuron.

[9] Koser D.E., Thompson A.J., Foster S.K., Dwivedy A., Pillai E.K., Sheridan G.K., Svoboda H., Viana M., Costa L.D.F., Guck J. (2016). Mechanosensing Is Critical for Axon Growth in the Developing Brain. Nat. Neurosci..

[10] Li Q.Y., Duan Y.W., Zhou Y.H., Chen S.X., Li Y.Y., Zang Y. (2022). Nlrp3-Mediated Piezo1 Upregulation in Acc Inhibitory Parvalbumin-Expressing Interneurons Is Involved in Pain Processing after Peripheral Nerve Injury. Int. J. Mol. Sci..

[11] Velasco-Estevez M., Mampay M., Boutin H., Chaney A., Warn P., Sharp A., Burgess E., Moeendarbary E., Dev K.K., Sheridan G.K. (2018). Infection Augments Expression of Mechanosensing Piezo1 Channels in Amyloid Plaque-Reactive Astrocytes. Front. Aging Neurosci..

[12] Velasco-Estevez M., Gadalla K.K.E., Linan-Barba N., Cobb S., Dev K.K., Sheridan G.K. (2020). Inhibition of Piezo1 Attenuates Demyelination in the Central Nervous System. Glia.

[13] Zhu J., Xian Q., Hou X., Wong K.F., Zhu T., Chen Z., He D., Kala S., Murugappan S., Jing J. (2023). The Mechanosensitive Ion Channel Piezo1 Contributes to Ultrasound Neuromodulation. Proc. Natl. Acad. Sci. USA.

[14] Velasco-Estevez M., Rolle S.O., Mampay M., Dev K.K., Sheridan G.K. (2020). Piezo1 Regulates Calcium Oscillations and Cytokine Release from Astrocytes. Glia.

[15] Jäntti H., Sitnikova V., Ishchenko Y., Shakirzyanova A., Giudice L., Ugidos I.F., Gómez-Budia M., Korvenlaita N., Ohtonen S., Belaya I. (2022). Microglial Amyloid Beta Clearance Is Driven by Piezo1 Channels. J. Neuroinflammation.

[16] Harraz O.F., Klug N.R., Senatore A.J., Hill-Eubanks D.C., Nelson M.T. (2022). Piezo1 Is a Mecha nosensor Channel in Central Nervous System Capillaries. Circ. Res..

[17] Velasco-Estevez M., Koch N., Klejbor I., Caratis F., Rutkowska A. (2022). Mechanoreceptor Piezo1 Is Downregulated in Multiple Sclerosis Brain and Is Involved in the Maturation and Migration of Oligodendrocytes in Vitro. Front. Cell. Neurosci..

[18] Qiu Z., Guo J., Kala S., Zhu J., Xian Q., Qiu W., Li G., Zhu T., Meng L., Zhang R. (2019). The Mechanosensitive Ion Channel Piezo1 Significantly Mediates in Vitro Ultrasonic Stimulation of Neurons. IScience.

[19] Yoo S., Mittelstein D.R., Hurt R.C., Lacroix J., Shapiro M.G. (2022). Focused Ultrasound Excites Cortical Neurons Via Mechanosensitive Calcium Accumulation and Ion Channel Amplification. Nat. Commun..

[20] Johnstone A.F.M., Gross G.W., Weiss D.G., Schroeder O.H.-U., Gramowski A., Shafer T.J. (2010). Microelectrode Arrays: A Physiologically Based Neurotoxicity Testing Platform for the 21st Century. Neurotoxicology.

[21] Novellino A., Scelfo B., Palosaari T., Price A., Sobanski T., Shafer T.J., Johnstone A.F.M., Gross G.W., Gramowski A., Schroeder O. (2011). Development of Micro-Electrode Array Based Tests for Neurotoxicity: Assessment of Interlaboratory Reproducibility with Neuroactive Chemicals. Front. Neuroeng..

[22] Parenti C., Turnaturi R., Aricò G., Gramowski-Voß A., Schroeder O.H.-U., Marrazzo A., Prezzavento O., Ronsisvalle S., Scoto G.M., Ronsisvalle G. (2013). The Multitarget Opioid Ligand Lp1’s Effects in Persistent Pain and in Primary Cell Neuronal Cultures. Neuropharmacology.

[23] Accardi M.V., Pugsley M.K., Forster R., Troncy E., Huang H., Authier S. (2016). The Emerging Role of in Vitro Electrophysiological Methods in Cns Safety Pharmacology. J. Pharmacol. Toxicol. Methods.

[24] Charkhkar H., Frewin C., Nezafati M., Knaack G.L., Peixoto N., Saddow S.E., Pancrazio J.J. (2014). Use of Cortical Neuronal Networks for in Vitro Material Biocompatibility Testing. Biosens. Bioelectron..

[25] Atmaramani R., Chakraborty B., Rihani R.T., Usoro J., Hammack A., Abbott J., Nnoromele P., Black B.J., Pancrazio J.J., Cogan S.F. (2020). Ruthenium Oxide Based Microelectrode Arrays for in Vitro and in Vivo Neural Recording and Stimulation. Acta Biomater..

[26] Gross G.W., Wen W.Y., Lin J.W. (1985). Transparent Indium-Tin Oxide Electrode Patterns for Extracellular, Multisite Recording in Neuronal Cultures. J. Neurosci. Methods.

[27] Gross G.W., Rhoades B.K., Azzazy H.M.E., Wu M.-C. (1995). The Use of Neuronal Networks on Multielectrode Arrays as Biosensors. Biosens. Bioelectron..

[28] Gnanasambandam R., Ghatak C., Yasmann A., Nishizawa K., Sachs F., Ladokhin A.S., Sukharev S.I., Suchyna T.M. (2017). Gsmtx4: Mechanism of Inhibiting Mechanosensitive Ion Channels. Biophys. J..

[29] Syeda R., Xu J., Dubin A.E., Coste B., Mathur J., Huynh T., Matzen J., Lao J., Tully D.C., Engels I.H. (2015). Chemical Activation of the Mechanotransduction Channel Piezo1. eLife.

[30] Botello-Smith W.M., Jiang W., Zhang H., Ozkan A.D., Lin Y.C., Pham C.N., Lacroix J.J., Luo Y. (2019). A Mechanism for the Activation of the Mechanosensitive Piezo1 Channel by the Small Molecule Yoda1. Nat. Commun..

[31] Xiang G., Pan L., Huang L., Yu Z., Song X., Cheng J., Xing W., Zhou Y. (2007). Microelectrode Array-Based System for Neuropharmacological Applications with Cortical Neurons Cultured in Vitro. Biosens. Bioelectron..

[32] Buzsáki G., Mizuseki K. (2014). The Log-Dynamic Brain: How Skewed Distributions Affect Network Operations. Nat. Rev. Neurosci..

[33] Valsky D., Grosberg S.H., Israel Z., Boraud T., Bergman H., Deffains M. (2020). What Is the True Discharge Rate and Pattern of the Striatal Projection Neurons in Parkinson’s Disease and Dystonia?. eLife.

[34] Moaddab M., Ray M.H., McDannald M.A. (2021). Ventral Pallidum Neurons Dynamically Signal Relative Threat. Commun. Biol..

[35] Paulk A.C., Yang J.C., Cleary D.R., Soper D.J., Halgren M., O’donnell A.R., Lee S.H., Ganji M., Ro Y.G., Oh H. (2021). Microscale Physiological Events on the Human Cortical Surface. Cereb. Cortex.

[36] Li D.L., Ma Z.Y., Fu Z.J., Ling M.Y., Yan C.Z., Zhang Y. (2014). Glibenclamide Decreases Atp-Induced Intracellular Calcium Transient Elevation Via Inhibiting Reactive Oxygen Species and Mitochondrial Activity in Macrophages. PLoS ONE.

[37] Lackovic J., Jeevakumar V., Burton M., Price T.J., Dussor G. (2023). Peroxynitrite Contributes to Behavioral Responses, Increased Trigeminal Excitability, and Changes in Mitochondrial Function in a Preclinical Model of Migraine. J. Neurosci..

[38] Rotordam M.G., Fermo E., Becker N., Barcellini W., Brüggemann A., Fertig N., Egée S., Rapedius M., Bianchi P., Kaestner L. (2019). A Novel Gain-of-Function Mutation of Piezo1 Is Functionally Affirmed in Red Blood Cells by High-Throughput Patch Clamp. Haematologica.

[39] Wagenaar D.A., Madhavan R., Pine J., Potter S.M. (2005). Controlling Bursting in Cortical Cultures with Closed-Loop Multi-Electrode Stimulation. J. Neurosci..

[40] Bowman C.L., Gottlieb P.A., Suchyna T.M., Murphy Y.K., Sachs F. (2007). Mechanosensitive Ion Channels and the Peptide Inhibitor Gsmtx-4: History, Properties, Mechanisms and Pharmacology. Toxicon.

[41] Henze D.A., Borhegyi Z., Csicsvari J., Mamiya A., Harris K.D., Buzsaki G. (2000). Intracellular Features Predicted by Extracellular Recordings in the Hippocampus in Vivo. J. Neurophysiol..

[42] Etzlaff C., Okujeni S., Egert U., Wörgötter F., Butz M. (2010). Self-Organized Criticality in Developing Neuronal Networks. PLoS Comput. Biol..

[43] Massobrio P., Pasquale V., Martinoia S. (2015). Self-Organized Criticality in Cortical Assemblies Occurs in Concurrent Scale-Free and Small-World Networks. Sci. Rep..

[44] DeMarse T., Cadotte A., Douglas P., He P., Trinh V. (2004). Computation within Cultured Neural Networks. Proceedings of the 26th Annual International Conference of the IEEE Engineering in Medicine and Biology Society.

[45] Pancrazio J.J., Gopal K., Keefer E.W., Gross G.W. (2014). Botulinum Toxin Suppression of Cns Network Activity in Vitro. J. Toxicol..

[46] Morefield S.I., Keefer E.W., Chapman K.D., Gross G.W. (2000). Drug Evaluations Using Neuronal Networks Cultured on Microelectrode Arrays. Biosens. Bioelectron..

[47] Lacroix J.J., Botello-Smith W.M., Luo Y. (2018). Probing the Gating Mechanism of the Mechanosensitive Channel Piezo1 with the Small Molecule Yoda1. Nat. Commun..

[48] Fang X.-Z., Zhou T., Xu J.-Q., Wang Y.-X., Sun M.-M., He Y.-J., Pan S.-W., Xiong W., Peng Z.-K., Gao X.-H. (2021). Structure, Kinetic Properties and Biological Function of Mechanosensitive Piezo Channels. Cell Biosci..

[49] Wang Y.Y., Zhang H., Ma T., Lu Y., Xie H.Y., Wang W., Ma Y.H., Li G.H., Li Y.W. (2019). Piezo1 Mediates Neuron Oxygen-Glucose Deprivation/Reoxygenation Injury Via Ca(2+)/Calpain Signaling. Biochem. Biophys. Res. Commun..

[50] Zhang Q.-Y., Zhang Y.-Y., Xie J., Li C.-X., Chen W.-Y., Liu B.-L., Wu X.-A., Li S.-N., Huo B., Jiang L.-H. (2014). Stiff Substrates Enhance Cultured Neuronal Network Activity. Sci. Rep..

[51] Bray D. (1979). Mechanical Tension Produced by Nerve Cells in Tissue Culture. J. Cell Sci..

[52] Franze K., Gerdelmann J., Weick M., Betz T., Pawlizak S., Lakadamyali M., Bayer J., Rillich K., Gögler M., Lu Y.-B. (2009). Neurite Branch Retraction Is Caused by a Threshold-Dependent Mechanical Impact. Biophys. J..

[53] Kerstein P.C., Jacques-Fricke B.T., Rengifo J., Mogen B.J., Williams J.C., Gottlieb P.A., Sachs F., Gomez T.M. (2013). Mechanosensitive Trpc1 Channels Promote Calpain Proteolysis of Talin to Regulate Spinal Axon Outgrowth. J. Neurosci..

[54] Saccher M., Kawasaki S., Onori M.P., van Woerden G.M., Giagka V., Dekker R. (2022). Focused Ultrasound Neuromodulation on a Multiwell Mea. Bioelectron. Med..

[55] Lewis A.H., Cui A.F., McDonald M.F., Grandl J. (2017). Transduction of Repetitive Mechanical Stimuli by Piezo1 and Piezo2 Ion Channels. Cell Rep..

[56] Coste B., Xiao B., Santos J.S., Syeda R., Grandl J., Spencer K.S., Kim S.E., Schmidt M., Mathur J., Dubin A.E. (2012). Piezo Proteins Are Pore-Forming Subunits of Mechanically Activated Channels. Nature.

[57] Gold C., Henze D.A., Koch C. (2007). Using Extracellular Action Potential Recordings to Constrain Compartmental Models. J. Comput. Neurosci..

[58] Boada M.D., Ririe D.G., Eisenach J.C. (2017). Post-Discharge Hyperpolarization Is an Endogenous Modulatory Factor Limiting Input from Fast-Conducting Nociceptors (Ahtmrs). Mol. Pain.

[59] Mulica P., Grunewald A., Pereira S.L. (2021). Astrocyte-Neuron Metabolic Crosstalk in Neurodegeneration: A Mitochondrial Perspective. Front. Endocrinol..

[60] Yang Y., Vidensky S., Jin L., Jie C., Lorenzini I., Frankl M., Rothstein J.D. (2011). Molecular Comparison of Glt1+ and Aldh1l1+ Astrocytes in Vivo in Astroglial Reporter Mice. Glia.

